# Implicit Talker Training Improves Comprehension of Auditory Speech in Noise

**DOI:** 10.3389/fpsyg.2017.01584

**Published:** 2017-09-14

**Authors:** Jens Kreitewolf, Samuel R. Mathias, Katharina von Kriegstein

**Affiliations:** ^1^Department of Psychology, University of Lübeck Lübeck, Germany; ^2^Max Planck Institute for Human Cognitive and Brain Sciences Leipzig, Germany; ^3^Department of Psychiatry, Yale University, New Haven CT, United States; ^4^Department of Psychology, Humboldt University of Berlin Berlin, Germany

**Keywords:** implicit training, voice learning, talker familiarity, familiarity benefit, speech comprehension, speech-reception thresholds, speech-in-noise task

## Abstract

Previous studies have shown that listeners are better able to understand speech when they are familiar with the talker’s voice. In most of these studies, talker familiarity was ensured by explicit voice training; that is, listeners learned to identify the familiar talkers. In the real world, however, the characteristics of familiar talkers are learned incidentally, through communication. The present study investigated whether speech comprehension benefits from implicit voice training; that is, through exposure to talkers’ voices without listeners explicitly trying to identify them. During four training sessions, listeners heard short sentences containing a single verb (e.g., “he writes”), spoken by one talker. The sentences were mixed with noise, and listeners identified the verb within each sentence while their speech-reception thresholds (SRT) were measured. In a final test session, listeners performed the same task, but this time they heard different sentences spoken by the familiar talker and three unfamiliar talkers. Familiar and unfamiliar talkers were counterbalanced across listeners. Half of the listeners performed a test session in which the four talkers were presented in separate blocks (blocked paradigm). For the other half, talkers varied randomly from trial to trial (interleaved paradigm). The results showed that listeners had lower SRT when the speech was produced by the familiar talker than the unfamiliar talkers. The type of talker presentation (blocked vs. interleaved) had no effect on this familiarity benefit. These findings suggest that listeners implicitly learn talker-specific information during a speech-comprehension task, and exploit this information to improve the comprehension of novel speech material from familiar talkers.

## Introduction

Natural speech provides the listener with a wealth of information, not only about what is said, but also about the identity of the talker. The acoustic features used to recognize talkers, such as pitch, timbre, and the acoustic effect of articulatory style, introduce large amounts of variability into the speech signal (reviewed by Nygaard, 2005). Nevertheless, listeners understand speech from a variety of different talkers with apparent ease (Peterson and Barney, 1952; Abramson and Cooper, 1959). This basic observation suggests that the ability to understand speech from different talkers involves active processing of talker information during speech comprehension (reviewed by Nusbaum and Magnuson, 1997). Indeed, it has been shown that talker-specific characteristics are perceived and memorized along with the speech message (Palmeri et al., 1993; Pisoni, 1993; Bradlow et al., 1999), and that familiarity with a talker’s voice shapes the perception of speech signals (e.g., Eisner and McQueen, 2005; for a review, see Cutler et al., 2010). One potential benefit of this integrated processing of talker and speech information is enhanced speech comprehension for familiar talkers (Nygaard et al., 1994; Magnuson et al., 1995; Nygaard and Pisoni, 1998; Yonan and Sommers, 2000; Newman and Evers, 2007; Levi et al., 2011; Johnsrude et al., 2013). In the following, we refer to this effect as the “familiarity benefit.”

Previous studies investigating the familiarity benefit have induced talker familiarity via *explicit voice training* (Nygaard et al., 1994; Magnuson et al., 1995; Nygaard and Pisoni, 1998; Levi et al., 2011). For example, in the study by Nygaard and Pisoni (1998), one group of listeners was trained to identify a set of 10 talkers via voice-name associations. The authors found that, following training, this group of listeners was better able to comprehend speech produced by the 10 talkers than a second group of listeners who did not have prior experience with the talkers. It could be argued that such explicit voice training is somewhat unrealistic because, in the real world, we are exposed to acoustic talker information while, most of the time, being actively engaged in speech comprehension. This means that we rarely learn acoustic talker information explicitly, but rather incidentally while understanding speech. Thus, real-world voice training can be considered to be a form of implicit (or “task-irrelevant”) training (for reviews, see Cleeremans et al., 1998; DeKeyser, 2008; Seitz and Watanabe, 2009).

In the present study, we considered whether implicit voice training elicits a familiarity benefit. Although many learning studies use explicit training (e.g., reviewed by DeKeyser, 2008), implicit training is often successful for various types of material, including formant transitions (Seitz et al., 2010) and phonetic contrasts (Vlahou et al., 2012). Whether this is also the case for the familiarity benefit is currently unknown. In one study, Yonan and Sommers (2000) claimed to show a familiarity benefit after implicit voice training. However, their study included an assessment of voice recognition prior to speech recognition testing; it is therefore unclear whether the familiarity benefit was due to wholly implicit voice training, or a combination of explicit and implicit training. In another study, Burk et al. (2006) used implicit voice training, but failed to show a familiarity benefit. Surprisingly, their listeners were actually worse at understanding speech from the familiar talker than the unfamiliar talkers. However, Burk et al. (2006) trained all of their listeners on the same talker. Thus, any effect of talker familiarity may have been masked by talker-specific effects, such as lower intelligibility of the familiar talker compared to the unfamiliar talkers.

**FIGURE 1:**
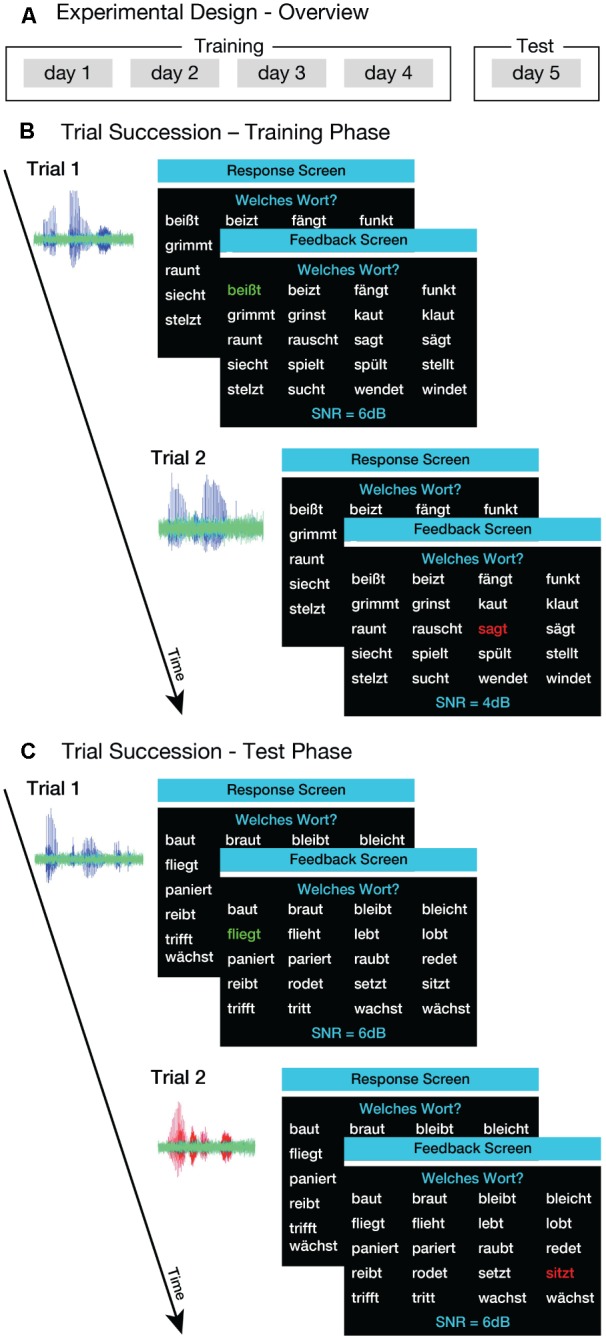
Experimental procedure. **(A)** Overview of the experimental design. The training phase comprised four sessions on four consecutive days. On the 5th day, listeners completed a test phase. **(B)** Example of trial succession in the training phase. Listeners heard sentences that were produced by one of four male talkers. The sentences (blue waveforms) were masked with speech-shaped noise (green). Listeners were asked to select the verb that was present in the sentence from the 20 options displayed on the screen (‘Response Screen’). After each trial, feedback was provided in terms of green or red coloring of the selected verb (‘Feedback Screen’). **(C)** Example of trial succession in the test phase. Listeners heard sentences produced by the same talker that was presented during the training phase (familiar talker; blue waveform) and by three novel talkers (unfamiliar talkers; red waveform). The general procedure and task of the test phase was the same as in the training phase.

Here, we employed a purely implicit voice-training paradigm. During the training, listeners heard sentences produced by one talker (familiar talker), while performing a speech-in-noise comprehension task; thus, their attention was never drawn to the talker’s identity (**Figure 1A**, ‘Training’; **Figure 1B**). After the training, listeners performed the same task, but this time sentences were produced by the familiar talker as well as by three unfamiliar talkers (**Figure 1A**, ‘Test’; **Figure 1C**). Importantly, we controlled for differences across talkers by counterbalancing which talker was the familiar talker at the group level. We hypothesized that listeners would benefit from the implicit voice training. If this hypothesis was correct, listeners would attain lower speech-reception thresholds (SRTs) for the familiar talker than for the unfamiliar talkers in the test phase.

Another aim of the present study was to explore the influence of the design of the test phase on the familiarity benefit. To do this, we used two slightly different paradigms. For half of the listeners, speech stimuli from the four talkers (one familiar, three unfamiliar) were presented in separate blocks of trials during the test phase (blocked paradigm). For the other half, the test phase comprised blocks of trials in which the talker varied randomly from trial to trial (interleaved paradigm). Previous research has shown that blocked relative to mixed presentation of talkers improves speech comprehension (Mullennix et al., 1989; Best et al., 2008; Kitterick et al., 2010; Bent and Holt, 2013). We therefore hypothesized that the listeners would benefit from blocked talker presentation. If this hypothesis was correct, listeners in the blocked paradigm would attain lower SRTs than those in the interleaved paradigm. Furthermore, the manipulation of paradigm allowed us to investigate whether blocked talker presentation provides better access to the acoustic talker information learned during training. If this is the case, listeners in the blocked paradigm should have a larger familiarity benefit than those in the interleaved paradigm.

## Materials and Methods

### Listeners

Twenty-four listeners (16 females; mean age 25.6 years; age range 21–30 years) were included in the study. All of the listeners were university students and native German speakers. None of them had prior experience with the talkers used in this study, or had any history of neurological or psychiatric disorder. All of them had normal hearing [less than 20 dB hearing level (HL)] in both ears, as assessed with pure-tone audiometry for frequencies in octave steps between 0.25 and 8 KHz (Micromate 304, Madsen Electronics, Denmark). In addition to the 24 normal-hearing listeners included in the study, one more listener was tested, but showed HLs that exceeded 20 dB HL and was therefore excluded from the experiment. Written informed consent was collected from all listeners according to procedures approved by the Research Ethics Committee of the University of Leipzig. Listeners were paid after completing the experiment.

### Stimuli

The stimuli were 200 short German sentences. Each sentence consisted of one noun and one verb. Each sentence started with *Er* [International Phonetic Alphabet (IPA): 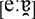; English: ‘he’]. The set of 200 verbs was made up of 100 minimal pairs – that is, for each verb in the set, there was another one that differed in a single phoneme (e.g., *Er schreibt.* vs. *Er schreit.* IPA: 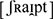 vs. 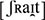; English: “He writes” vs. “He screams”). Four talkers, who were all male and native German speakers (mean age 26.8 years; age range 23–31 years), produced the complete set of sentences. The talkers were students of speech communication (*Sprechwissenschaften*) and received speech training as part of their studies. All talkers spoke Standard German without an obvious dialect. For the recordings, they were instructed to speak in neutral manner and in their natural tone of voice. Recordings were made in a sound-attenuating chamber (IAC – I200 series, Winchester, United Kingdom) with a resolution of 16 bits and at a sampling rate of 44.1 kHz using a cardioid condenser microphone (RØDE NT55, Silverwater, NSW, Australia). All stimuli were adjusted to the same root mean square (RMS) value using MATLAB (version 7.11, MathWorks, United States).

During the experiment, sentences were mixed with speech-shaped noise (i.e., white noise filtered to have the same long-term average spectrum as the average of all the speech stimuli) created on the fly using the fftfilt function implemented in the MATLAB signal processing toolbox. This procedure ensured that each token of speech-shaped noise was a different waveform and thus prevented listeners from learning regularities in the noise. Speech and noise sounds were matched in duration (mean duration = 890 ms; *SD* = 93 ms). Different signal-to-noise ratios (SNRs) were created by manipulating the sound level of the speech stimuli; the level of the noise was kept constant. The stimuli were delivered diotically through headphones (Sennheiser HD580, Wedemark, Germany) at about 65 dB SPL using a 16-bit digital-to-analog converter (Creative Sound Blaster Audigy 2 ZS, Jurong East, Singapore) at a sampling rate of 44.1 kHz and a pre-amplifier (Pro-Ject Head Box II, Vienna, Austria).

### Procedure

#### Training Phase

The experiment included a training phase and a test phase, summarized in **Figure 1**. The training phase comprised four sessions performed on consecutive days (**Figure 1A**, left). During training, listeners heard sentences spoken by one of the four talkers (i.e., the familiar talker). The choice of familiar talker was counterbalanced across listeners. Listeners heard one sentence from this talker per trial, while twenty verbs were displayed on the computer screen, together with the question *Welches Wort?* (English: ‘Which word?’), and the current SNR value (**Figure 1B**). Listeners were asked to click on the verb that was present in the sentence from these 20 options. After each trial, listeners received feedback in terms of green (correct) or red (incorrect) coloring of the selected verb (**Figure 1B**). The SNR was set initially to +6 dB and was manipulated using a weighted one-up one-down adaptive procedure that estimates SRTs corresponding to 75%-correct on the psychometric function (Kaernbach, 1991). For the first four reversals in the direction of the staircase, SNR was decreased by 2 dB following a correct response, and increased by 6 dB following an incorrect response. From the fifth reversal onward, the step sizes were 0.67 and 2 dB for down- and up-steps, respectively. The staircase was terminated after the 12th reversal, and the SRT was defined as the arithmetic mean of SNR values visited on all reversal trials after the fifth reversal. Listeners were instructed to decrease the SNR value as much as possible, and that they would gain an additional monetary reward on each day of training if their average SRT was below -5 dB.

One hundred and thirty-two of the 200 sentences were used as stimuli in the training phase (the remaining 68 sentences were reserved for the test phase; see below). In each block of trials (one block corresponding to one SRT measurement), 20 of the 132 sentences were used as the possible response options. The 20 response options were composed of 10 minimal pairs so that both members of a given pair were always present in the response set. For each trial, one sentence corresponding to 1 of the 20 response options was selected at random. A sentence could be used in more than one training block and in more than one of the four training sessions. Across all listeners and training sessions, there were on average 39.11 trials per block (*SD* = 6.28). Each of the 20 possible sentences was presented on average 1.99 times within a block (*SD* = 0.35) and on average 17.29 different sentences were presented within a block (*SD* = 1.45). Each training session contained 20 blocks, and lasted about 90 min.

#### Test Phase

The test phase was conducted on the 5th day of the study (**Figure 1A**, ‘Test’). The experimental procedure was identical to that used in the training phase, except for three differences: (i) the test stimuli were the remaining 68 sentences that had not been used in the training; (ii) the sentences were spoken by the same talker as in the training phase (familiar talker) as well as by three unfamiliar talkers; and (iii) for half of the listeners, the talker changed randomly from trial to trial within a block (see below). As in the training sessions, sentences were overlaid with speech-shaped noise and SRTs were measured using the same adaptive tracking procedure. The trial structure and task was the same as in the training phase (**Figure 1C**).

Half of the listeners (10 females; mean age 26.3 years; age range 22–30 years) performed one version of the test phase, in which all the sentences within a block were spoken by the same talker (blocked paradigm). Each talker was presented in five blocks, amounting to 20 blocks in the test phase. Identical verb displays were used for all talkers to ensure that differences in SRTs between talkers were not due to differences in the presented stimuli. The order of blocks was randomized with the restriction that all four talkers were presented in four consecutive blocks. Furthermore, we ensured that there was always a change in talker between two consecutive blocks.

The remaining half of the listeners (6 females; mean age 24.9 years; age range 21–30 years) performed another version of the test phase, in which the talker changed randomly from trial to trial within a block (interleaved paradigm). Within one block of the interleaved paradigm, SRTs for each of the four talkers were tracked independently, and a block ended only when the staircases of all four talkers reached 12 reversals. Due to randomization, this could result in more than 12 reversals for the staircases of some talkers. However, only the first 12 reversals per talker were analyzed. There were five blocks in the interleaved paradigm, resulting in 20 SRTs.

In both blocked and interleaved paradigms, as in the training phase, feedback was provided immediately after each trial (**Figure 1C**). Again, listeners could infer the difficulty level of the current trial from the SNR value displayed on the computer screen. Since four staircases (one per talker) were simultaneously tracked in the interleaved paradigm, the mean SNR value over all four staircases was presented instead. As in the training sessions, listeners could gain an additional monetary reward if their average SRT was below -5 dB. The test phase lasted about 100 min for each participant. On average, listeners received a total compensation of 62.58 €; (*SD* = 1.56 €;) for their participation in the training and test sessions.

### Data Analysis

Listeners’ SRTs were analyzed using linear mixed-effects models as implemented in R (R Core Team, 2017). Training and test-phase SRTs were analyzed separately. For both training- and test-phase SRTs, we followed an iterative model-fitting procedure: starting with the intercept-only models, first fixed- and then random-effects terms were added in a stepwise fashion; after each step, we fitted the model using maximum-likelihood estimation, and assessed the change in model fit using likelihood-ratio tests.

For the training-phase SRTs, we modeled the potential fixed effect of training session using forward difference coding; that is, the mean SRT for one training session was compared to the mean SRT for the subsequent training session. This coding scheme allowed us to assess whether the training-phase SRTs successively decreased over sessions. We used deviation coding for all predictors of the test-phase SRTs.

We derived *p*-values for individual model terms using the Satterthwaite approximation for degrees of freedom (Luke, 2017). To enhance the interpretability of non-significant effects, and to overcome some of the limitations associated with the comparably small sample size, we also calculated Bayes Factors (BFs) using the *BayesFactor* package in R. When comparing two statistical models, the BF indicates how many times more likely the observed data are under the more complex model compared to the simpler model. In accordance with Jeffreys (1939), a BF < 0.3 is interpreted as providing evidence in favor of the null hypothesis and a BF > 3 as evidence against it.

## Results

### Effect of Training

**FIGURE 2:**
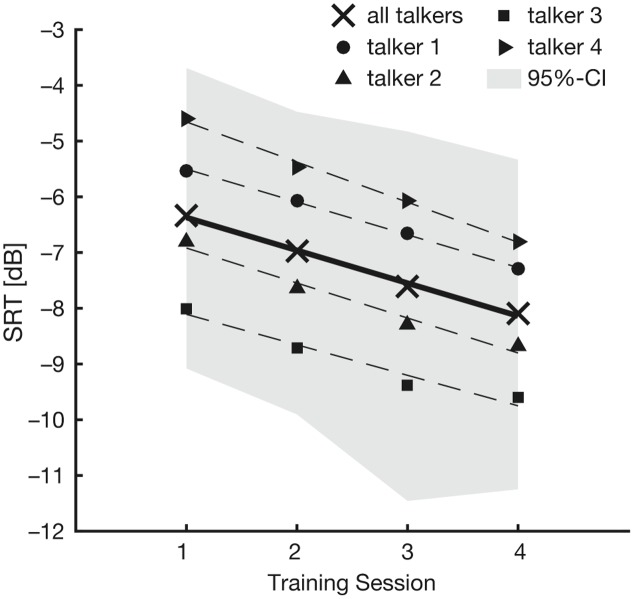
Results of the training phase. The figure shows mean SRTs in the four training sessions. Black crosses represent mean SRTs averaged across all talkers. Mean SRTs for each talker are coded by different symbols (talker 1: black circle; talker 2: black triangle, upward; talker 3: black square; talker 4: black triangle, rightward). Linear regression lines are plotted for mean SRTs across all talkers (solid line) and for each talker separately (dashed lines). The gray shaded area denotes the 95% confidence interval.

**Figure 2** shows the evolution of SRTs over training sessions separately for each of the four training talkers. Based on this figure, it seems that SRTs decreased over training sessions. The best-fitting linear mixed-effects model of the training-phase SRTs included training session as a fixed effect, and listener (‘subject’) as well as talker as random effects. There was a main effect of training session under this model [*F*_(3,1854.7)_ = 60.15; *p* < 0.001]. Importantly, there was a gradual decrease in the unstandardized coefficients (*b*) across the different levels of training session (session 1: *b* = 0.82 dB; session 2: *b* = 0.62 dB; session 3: *b* = 0.51 dB; all relative to session 4). These results confirmed our first observation from **Figure 2**, namely that the listeners’ comprehension of speech in noise gradually improved over training sessions.

A second observation from **Figure 2** is that SRTs appeared to vary considerably depending on the talker the listeners were trained on. To account for this variability, we included a random effect of talker. Compared to the simpler model (without a random effect of talker), the inclusion of talker significantly improved the model fit (χ^2^_1_ = 9.96; *p* = 0.002), and confirmed our second observation from **Figure 2**. To check whether the observed decrease in SRTs across training sessions depended on the training talker, we included random slopes for the by-talker effect of training session. This did not improve the model fit (χ^2^_9_ = 3.22; *p* = 0.95), suggesting that all listeners’ SRTs decreased over the course of the training similarly, irrespective of which talker they heard.

The variability in SRTs across talkers are evident in all training sessions, including the first one, suggesting that the talkers differed in intelligibility. We checked whether these talker-intelligibility differences could be explained by target-to-masker ratio (TMR; Gaudrain and Carlyon, 2013) or *f*0 range (Bradlow et al., 1996). Although speech sounds were adjusted to the same overall RMS prior to noise masking, it is possible that “instantaneous” TMR differed across talkers. For example, this can be the case when speech produced by one talker is more deeply modulated than speech produced by another talker. However, our acoustical analyses revealed that neither TMR nor *f*0 range could explain the differences in talker intelligibility (see Supplementary Material).

### Familiarity Benefit

**FIGURE 3:**
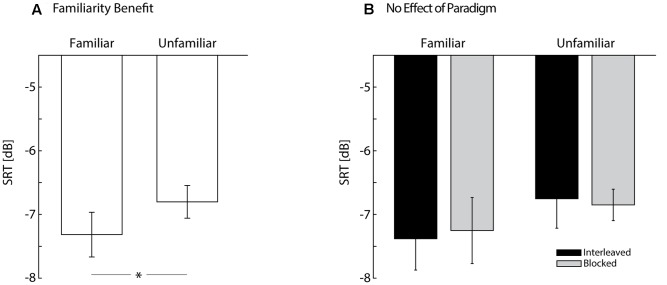
Results of the test phase. **(A)** Speech-reception thresholds (SRTs) are plotted separately for familiar and unfamiliar talkers. Note that lower SRTs indicate better performance in the speech-in-noise task. The asterisk indicates a significant main effect of talker familiarity (*p* = 0.024) with lower SRTs for familiar as compared to unfamiliar talkers (i.e., familiarity benefit). **(B)** SRTs are plotted separately for familiar and unfamiliar talkers as well as for listeners in the interleaved (black) and listeners in the blocked paradigm (gray). There was no significant difference in SRTs between listeners in the interleaved and listeners in the blocked paradigm (*p* = 0.88) and no significant interaction between paradigm and talker familiarity (*p* = 0.66). Bars show mean SRTs, error bars represent standard error of mean.

The results of the test phase are shown in **Figure 3**. The best-fitting model for the test-phase SRTs included talker familiarity (familiar vs. unfamiliar talkers) as a fixed effect, and listener as well as talker as random effects. The main effect of talker familiarity [*F*_(1,451)_ = 5.14; *p* = 0.024] confirmed our original hypothesis that implicit voice training leads to improved comprehension of speech from the familiar talker. On average, listeners were better at understanding speech in noise when the speech was produced by the familiar talker (-7.32 dB) than by the unfamiliar talkers (-6.80 dB) (**Figure 3A**). Similar to the analysis of training-phase SRTs, we included a random effect of talker (i.e., the four talkers each listener heard at test) to account for the variance in SRTs introduced by talkers. Compared to the simpler model (i.e., a model that included talker familiarity as a fixed effect and listener as the only random effect), the inclusion of talker significantly improved the model fit (χ^2^_1_ = 93.08; *p* < 0.001). We also included random slopes for the by-talker effect of talker familiarity, but this did not improve the model fit (χ^2^_2_ = 0.02; *p* = 0.99). Again, these results suggested differences in intelligibility across talkers. Importantly, however, the main effect of talker familiarity cannot be explained by intelligibility differences, because the best-fitting model included talker as a random effect. Furthermore, our study design ensured that the familiar talker was balanced across listeners, which means that, across all listeners, each talker was equally often the familiar talker.

### No Effect of Paradigm

Neither the inclusion of paradigm as a fixed effect (blocked vs. interleaved), nor the inclusion of the interaction between talker familiarity and paradigm as a fixed effect, improved the model fit compared to the simpler model (χ^2^_1_ = 0.03; *p* = 0.88; BF = 0.25, and χ^2^_1_ = 0.20; *p* = 0.66; BF = 0.17, respectively). This suggests that the listeners in the blocked and interleaved versions of the test phase attained similar SRTs, and that they benefited similarly from talker familiarity (**Figure 3B**). Furthermore, the BFs provide evidence in favor of the null hypotheses, indicating that the non-significant effects of paradigm were not due to the comparably small sample size (12 listeners participated in each of the two paradigms).

## Discussion

In the present study, we investigated whether listeners benefit from prior experience with a talker’s voice when understanding speech. In contrast to previous studies that used explicit voice training (Nygaard et al., 1994; Magnuson et al., 1995; Nygaard and Pisoni, 1998; Levi et al., 2011), we employed a training paradigm in which familiarity with a talker’s voice was induced incidentally through a speech-in-noise comprehension task. Such implicit voice training is similar to how we acquire knowledge about talker characteristics in the real world. The main result of the present study was that, listeners attained lower SRTs for speech produced by the familiar talker compared to the unfamiliar talkers (**Figure 3A**). The present study therefore showed that implicit voice training can confer a familiarity benefit. The familiarity benefit was not affected by the presentation of the talker (i.e., listeners in the interleaved and blocked paradigm benefited similarly from talker familiarity) (**Figure 3B**).

A previous study that used implicit voice training failed to find a familiarity benefit (Burk et al., 2006). All listeners in that experiment were trained using speech from just one talker. Such a design does not control for differences in intelligibility across talkers. Thus, the lack of familiarity benefit in the study by Burk et al. (2006) might have resulted from low intelligibility of the trained talker relative to the unfamiliar talkers. One could assume a similar result in the present study if all listeners would have been trained on one of the less intelligible talkers 1 and 4 (cf. **Figure 2**). Unlike the study by Burk et al. (2006), however, we controlled for effects of talker intelligibility by familiarizing an equal number of listeners with each of the four talkers.

Our results are consistent with a growing body of research suggesting that, in general, subjects learn irrelevant stimulus features (and are better able to discriminate those features later on) while performing a task on another stimulus feature (reviewed by Seitz and Watanabe, 2009). This implies that successful perceptual learning does not require the subjects to explicitly focus on the stimulus feature being learned. For example, Vlahou et al. (2012) showed that adult listeners can learn a phonetic contrast not found in their native language while performing an intensity-discrimination task. This training paradigm was just as successful as when listeners explicitly discriminated between phonetic categories. However, their results also showed that implicitly trained language skills did not generalize to novel acoustic input: listeners were not able to discriminate phonetic categories when sounds were produced by a talker who was not presented during training. The implicit voice training employed in the present study differed from typical training paradigms within the task-irrelevant learning framework. For example, we did not test whether listeners learned to discriminate the familiar talker from the unfamiliar talkers, to avoid contamination of the familiarity benefit by explicit focus on the talker identity (cf. Yonan and Sommers, 2000). Nevertheless, our findings provide further support for implicit perceptual learning and demonstrated that implicitly induced talker familiarity generalized to novel acoustic input: we showed that the familiarity benefit persists when listeners are presented with sentences they were not trained on. This is in line with previous reports of talker-specific adaptation in speech comprehension using different types of speech material (Bradlow and Pisoni, 1999) and noise (Bent et al., 2009; Van Engen, 2012). Our results suggest that even for implicit learning, the learned talker information is not restricted to instance-specific exemplars (Goldinger, 1998), but rather that listeners are able to acquire knowledge about the acoustic properties of a talker from a certain set of speech tokens and transfer this knowledge to novel tokens. Yet, the present study did not reveal what kind of talker-specific information the listeners learned via implicit voice learning. Listeners could have learned, for example, details about the talker’s vocal-tract and glottal-fold parameters (e.g., Baumann and Belin, 2010), articulatory style (e.g., Remez et al., 1997), or any combination of these features. One might speculate that the linguistic nature of our training procedure facilitated learning of talker-specific articulatory cues, whereas the explicit voice training employed in previous studies might have facilitated learning a more comprehensive set of voice-identity properties. However, whether this is indeed the case is impossible to find out with the present data set.

To date, little is known about the (neural) mechanisms underlying the familiarity benefit. It has been suggested that the effects of talker familiarity are based on amodal information about a talker’s articulatory style, because a familiarity benefit for auditory speech comprehension can be observed following training under purely visual conditions (Rosenblum et al., 2007). This would suggest that the familiarity benefit for auditory speech comprehension relies on the same, amodal mechanism, independent of the training condition; that is, implicit auditory training (present study), explicit auditory training (Nygaard et al., 1994; Magnuson et al., 1995; Nygaard and Pisoni, 1998; Levi et al., 2011) or even implicit visual training (Rosenblum et al., 2007). An alternative view is that the familiarity benefit relies on different mechanisms which are dependent on the modality and type of training. In this view, a familiarity benefit for auditory speech would be induced by functional interactions between brain areas in the left and right hemispheres that are sensitive to specific acoustic features of speech and talker (von Kriegstein et al., 2010; Kreitewolf et al., 2014). In contrast, the familiarity benefits for auditory speech induced by implicit visual training would be induced by a close interaction between auditory speech and visual areas that are sensitive to the visual talker-specific articulatory cues (von Kriegstein et al., 2008; Schall and von Kriegstein, 2014). In the present study, we induced talker familiarity through auditory-only training. Thus, one can speculate that our familiarity benefit was due to an enhanced communication between speech- and talker-sensitive brain areas.

The present study pertains to an important aspect of real-world talker familiarity – that is, listeners were familiarized with a talker’s voice through a speech comprehension task rather than through explicit talker identification. Yet, voice learning in the real world provides the listeners with more and qualitatively different information about the talker than voice learning under laboratory conditions. The effects of real-world voice learning might therefore be much larger than the relatively small familiarity benefit observed in the present study; this might be especially the case when the talker is personally familiar (Magnuson et al., 1995; Newman and Evers, 2007; Johnsrude et al., 2013). Furthermore, in real-world communication, listeners are rarely exposed to a talker in one modality only, but rather acquire talker familiarity through audio–visual exposure. It is therefore likely that the real-world familiarity benefit relies on a combination of modality-dependent and modality-independent mechanisms. To what extent real-world talker familiarity relies on either mechanism is, however, an open question.

## Ethics Statement

All subjects gave written informed consent in accordance with the Declaration of Helsinki. The study was approved by the Research Ethics Committee of the University of Leipzig.

## Author Contributions

JK and SM designed the experiment. JK conducted the experiment. JK, SM, and KvK performed the data analysis and interpretation. All authors contributed to the manuscript.

## Conflict of Interest Statement

The authors declare that the research was conducted in the absence of any commercial or financial relationships that could be construed as a potential conflict of interest.
