# Supplementary material for: Implicit Talker Training Improves Comprehension of Auditory Speech in Noise
Source: Front Psychol. 2017 Sep 14;8:1584. doi: 10.3389/fpsyg.2017.01584 (PMC5603660; doi:10.3389/fpsyg.2017.01584)
Supplement: Supplementary file 1 [file Supplementary_material.DOCX]

Supplementary Material

Implicit Talker Training Improves Comprehension of Auditory Speech in Noise

Jens Kreitewolf*, Samuel R. Mathias, Katharina von Kriegstein

*** Correspondence:** Jens Kreitewolf: jens.kreitewolf@uni-luebeck.de

# Supplementary Results: Talker intelligibility

To investigate the acoustic basis for the talker intelligibility differences during training, we checked whether energetic masking or the *f*0 range was different across the four talkers. To do this, we first evaluated the target-to-masker ratio (TMR) on 40 ms chunks of the 200 sentences from each talker mixed with speech-shaped noise at an SNR of 0 dB (Gaudrain and Carlyon, 2013). A Kruskal-Wallis test revealed that TMR was significantly different across talkers (χ(3) = 44.68, p < 0.001). Post-hoc Mann-Whitney U-tests, however, showed that the TMR was not higher for the more intelligible (talker 2 and 3) than the less intelligible talkers (talker 1 and 4) (Suppl. Fig. 1 A-D). Instead, TMR was significantly higher in talker 2 and talker 4 than in talker 1 and talker 3 (talker 1 vs. talker 2: U = 9.13×10^6^, p < 0.001; talker 1 vs. talker 4: U = 9.69×10^6^, p < 0.001; talker 2 vs. talker 3: U = 8.29×10^6^, p < 0.01; talker 3 vs. talker 4: U = 8.93×10^6^, p < 0.001; all other comparisons were not significant) (Suppl. Fig. 1; Suppl. Tab. 1). The summary TMR statistics were, however, very similar for all talkers (Suppl. Fig. 1 E) and the significant differences in TMR across talkers are probably due to the large sample size (there were between 3,946 and 4,748 TMR values per talker). In a second step, we checked whether talker-specific *f*0 range could account for differences in talker intelligibility (Bradlow et al., 1996). A one-way ANOVA revealed significant differences in *f*0 range across talkers (F_(3,766)_ = 40.83, p < 0.001). Post-hoc t-tests showed that the *f*0 range was larger for talker 3 than for any of the other talkers (talker 3 vs. 1: t(382) = 8.47, p < 0.001; talker 3 vs. 2: t(381) = 9.08, p < 0.001; talker 3 vs. 4: t(370) = 11.35, p < 0.001; all other comparisons were not significant) (Suppl. Fig. 2 Fig; Suppl. Tab. 1). As for TMR, talker-specific *f*0 range did not occur in the same direction as differences in talker intelligibility.

# Supplementary Figures and Tables

## Supplementary Figure 1

**
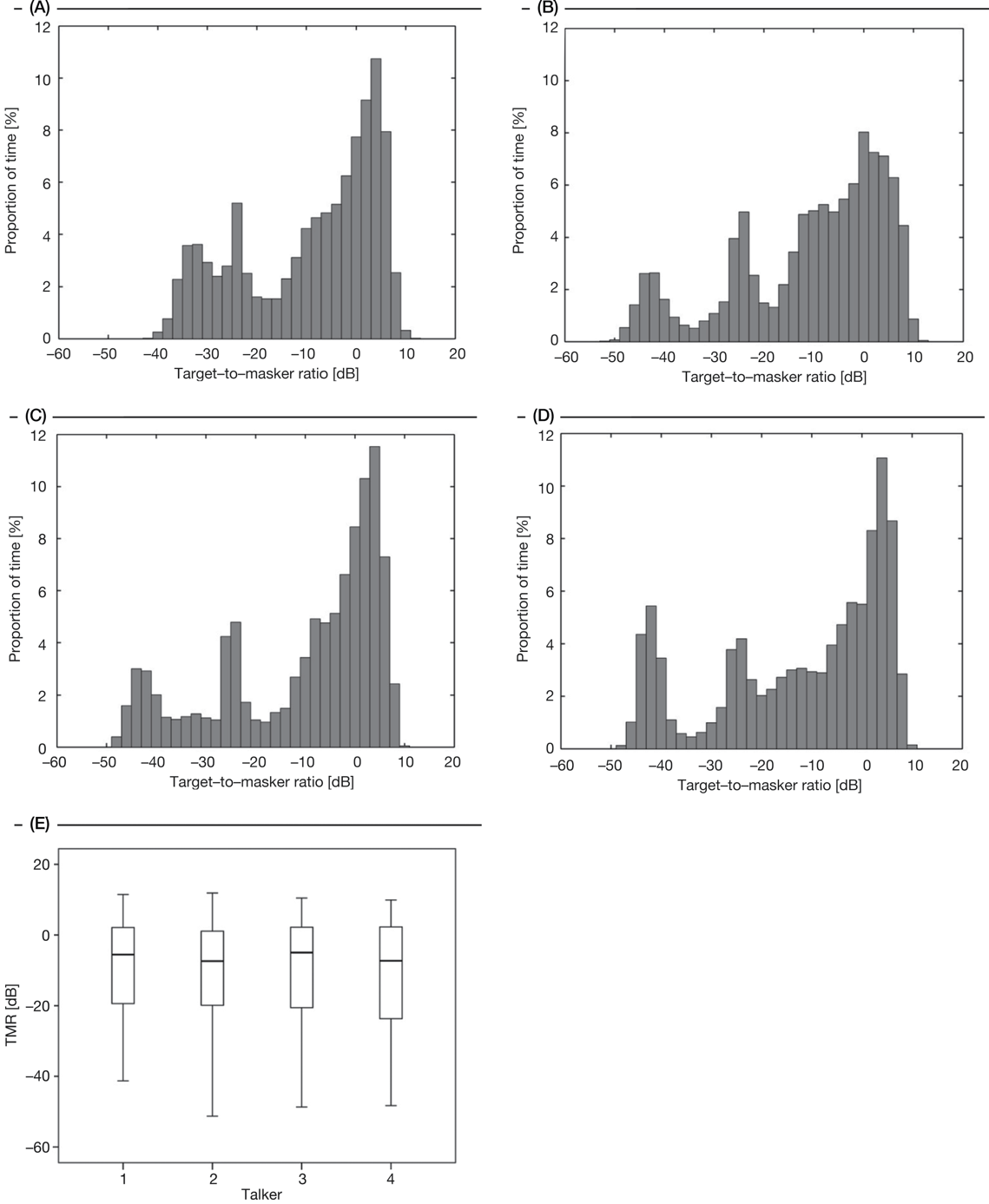
**

**Supplementary Figure 1.**

**Talker-specific target-to-masker ratio (TMR).** (A–D) Distributions of the TMR evaluated on 40 ms chunks of 200 target sentences (per talker) mixed with speech-shaped noise at an SNR of 0 dB. TMR distributions are shown separately for talker 1 (A), talker 2 (B), talker 3 (C), and talker 4 (D). (E) Boxplot of talker-specific TMR.

## Supplementary Figure 2

**
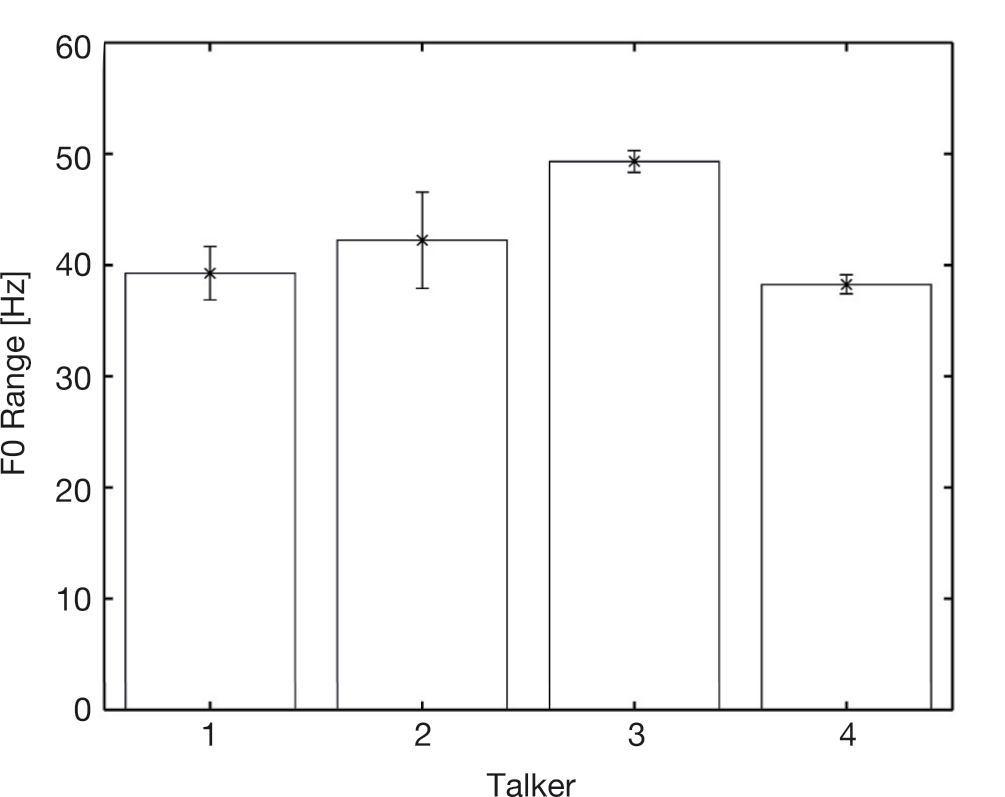
**

**Supplementary Figure 2.**

**Talker-specific *f*0 range.** Talker-specific *f*0 range calculated from 200 sentences (per talker). Bars represent means; error bars show standard error of mean.

## Supplementary Table 1

**Results of acoustical analyses.** Geometric mean f0 and f0 range (in Hz) was averaged across 200 sentences for each talker. Standard deviations are displayed in parentheses. Target-to-masker ratio (TMR) was evaluated on 40 ms chunks of 200 target sentences for each talker. Talker-specific median TMRs are shown with 95-% confidence intervals in parentheses.

| Talker | *f*0 mean  [Hz] | *f*0 range  [Hz] | TMR  [dB] |
| --- | --- | --- | --- |
| 1 | 85.25  (4.72) | 36.67  (13.65) | -5.55  (42.70) |
| 2 | 79.63  (4.32) | 36.25  (13.00) | -7.39  (52.50) |
| 3 | 124.26  (4.48) | 47.12  (10.12) | -4.95  (51.49) |
| 4 | 91.00  (2.74) | 35.96  (8.73) | -7.30  (51.31) |

## Appendix: List of Sentences

| Er baut. | Er fängt. | Er hängt. | Er knebelt. |
| --- | --- | --- | --- |
| Er beißt. | Er fehlt. | Er hebelt. | Er knobelt. |
| Er beizt. | Er feilt. | Er heftet. | Er kreischt. |
| Er beugt. | Er ficht. | Er herrscht. | Er kreist. |
| Er biegt. | Er fischt. | Er herzt. | Er lärmt. |
| Er bietet. | Er fliegt. | Er hinkt. | Er lebt. |
| Er bittet. | Er flieht. | Er hobelt. | Er leidet. |
| Er bleibt. | Er frisiert. | Er hockt. | Er leitet. |
| Er bleicht. | Er fritiert. | Er hofft. | Er lernt. |
| Er braut. | Er fühlt. | Er holt. | Er liebt. |
| Er denkt. | Er füllt. | Er jobbt. | Er liegt. |
| Er döst. | Er funkt. | Er joggt. | Er liest. |
| Er düngt. | Er grimmt. | Er kämmt. | Er lobt. |
| Er düst. | Er grinst. | Er kämpft. | Er lügt. |
| Er fährt. | Er haftet. | Er kaut. | Er mahnt. |
| Er fällt. | Er hält. | Er klaut. | Er meint. |
| Er nickt. | Er raucht. | Er saugt. | Er schnauft. |
| Er nippt. | Er räumt. | Er saust. | Er schneidet. |
| Er nutzt. | Er raunt. | Er schätzt. | Er schnippt. |
| Er nützt. | Er rauscht. | Er schaut. | Er schnitzt. |
| Er packt. | Er redet. | Er scheidet. | Er schraubt. |
| Er paniert. | Er reibt. | Er scheut. | Er schreibt. |
| Er pariert. | Er reicht. | Er schiebt. | Er schützt. |
| Er parkt. | Er reift. | Er schielt. | Er schwankt. |
| Er patzt. | Er reimt. | Er schießt. | Er schweigt. |
| Er poliert. | Er reist. | Er schläft. | Er schweißt. |
| Er posiert. | Er reizt. | Er schlägt. | Er schwenkt. |
| Er prahlt. | Er riecht. | Er schleckt. | Er schwimmt. |
| Er prallt. | Er rodet. | Er schleicht. | Er schwindelt. |
| Er putzt. | Er ruft. | Er schleift. | Er schwindet. |
| Er rächt. | Er ruht. | Er schleppt. | Er schwingt. |
| Er rast. | Er sagt. | Er schnappt. | Er schwitzt. |
| Er raubt. | Er sägt. | Er schnaubt. | Er senkt. |
| Er setzt. | Er stelzt. | Er tauscht. | Er wäscht. |
| Er siecht. | Er stiehlt. | Er tippt. | Er weilt. |
| Er siegt. | Er stockt. | Er tränkt. | Er weint. |
| Er sieht. | Er stoppt. | Er trennt. | Er wendet. |
| Er sinkt. | Er strafft. | Er trifft. | Er wertet. |
| Er sitzt. | Er straft. | Er trimmt. | Er windet. |
| Er spannt. | Er streckt. | Er trinkt. | Er winkt. |
| Er speist. | Er streicht. | Er tritt. | Er wirbt. |
| Er speit. | Er streikt. | Er verzeiht. | Er wirkt. |
| Er spickt. | Er strickt. | Er verzieht. | Er wischt. |
| Er spielt. | Er stutzt. | Er wachst. | Er wühlt. |
| Er spinnt. | Er stützt. | Er wächst. | Er würgt. |
| Er sprengt. | Er sucht. | Er wählt. | Er würzt. |
| Er springt. | Er tankt. | Er wandelt. | Er zählt. |
| Er spuckt. | Er tanzt. | Er wandert. | Er zaubert. |
| Er spült. | Er tappt. | Er wankt. | Er zaudert. |
| Er stellt. | Er taucht. | Er wartet. | Er zielt. |
